# Giant Two-photon Absorption in Circular Graphene Quantum Dots in Infrared Region

**DOI:** 10.1038/srep33260

**Published:** 2016-09-15

**Authors:** Xiaobo Feng, Zhisong Li, Xin Li, Yingkai Liu

**Affiliations:** 1 School of Physics and Electronic Information, Yunnan Normal University, Kunming 650500, China

## Abstract

We investigate theoretically the two-photon absorption (TPA) for circular graphene quantum dots (GQDs) with the edge of armchair and zigzag on the basis of electronic energy states obtained by solving the Dirac-Weyl equation numerically under finite difference method. The expressions for TPA cross section are derived and the transition selection rules are obtained. Results reveal that the TPA is significantly greater in GQDs than conventional semiconductor QDs in infrared spectrum (2–6 um) with a resonant TPA cross section of up to 10^11 ^GM. The TPA peaks are tuned by the GQDs’ size, edge and electron relaxation rate.

The unique structure of graphene, which is composed of a one-atom-thick two-dimensional honeycomb lattice of carbon atoms, contributes to its extraordinary physical and chemical properties, such as high intrinsic mobility, thermal stability, and electrical conductivity^1^,^2^,^3^. This attracted intense attention on new fundamental physics and promising applications in nanoelectronics. However, graphene is a zero-bandgap semiconductor, which limits its electronic and opto-electronic applications^4^. A bandgap, however, can be engineered into graphene quantum dots (GQDs) due to quantum confinement and edge effect^5^,^6^. Also, GQDs have advantages over conventional semiconductor QDs in terms of low toxicity, reduced photo bleaching, good solubility and high biocompatibility. Recent advances in chemical synthesis have enabled the achievement of GQDs with different sizes and shapes. The most common shape is circular, triangle and hexagon^7^,^8^. As we all know that quantum dot structures have been widely used as optical switches, bio-labels, biosensors and imaging agents through multi-photon absorption, due to the considerable enhancement of their nonlinear response caused by quantum size effects^9^,^10^,^11^. It is predicted that GQDs are also of rich nonlinear optical properties result from its unique structure as well as quantum confinement and edge effect. Therefore, GQDs have great potentials in light emitting, biomolecular sensing and cellular bioimaging applications^12^,^13^,^14^. In these applications, two-photon absorption (TPA) is an effective nonlinear optical process in laser excitation, owing to the advantages of longer excitation wavelength, deeper penetration depth and higher spatial resolution^15^.

In our previous research, we obtained both experimentally and theoretically that the TPA coefficient in bilayer graphene is as high as 10^5 ^cm/MW in the visible and infrared region^16^. As far as the optical properties of GQDs are concerned, most researches focused on linear optical properties. J. Peng *et al*. experimentally measured both the linear absorption spectra range from UV to visible and PL spectra of several different sized GQDs^17^. It is reported that the green upconversion photoluminescence of the uniform graphene oxide quantum disks (diameter = 4 nm) at ~563 nm was observed by a femto-second Ti: sapphire pulse laser^18^. Also, there are several measurements on TPA in carbon-related quantum dots. X. Zhang *et al*. conducted the two-photon fluorescence imaging of cellular nucleus in single-layered graphitic-C_3_N_4_ quantum dots, and it is measured that the TPA cross section of g- C_3_N_4_ in aqueous suspension has a maximum up to 28000 GM (1 GM = 10^−50^ cm^4^s/photon) at 750 nm^19^. It is reported that the carbon nanodots show quantum yields up to 22% and a high two-photon absorption cross section up to 2000 GM^20^. Actually, we have theoretically calculated the two-photon absorption coefficient in 4nm-sized circular graphene quantum dot, which is as high as 10^5 ^cm/MW^21^. However, in that calculation we employed the infinite mass boundary condition (IMBC), which ignored the effect of the quantum dots’ edge.

In this paper, we have deduced the expressions for TPA cross section for circular GQDs with edge of both armchair and zigzag on the basis of electronic energy states obtained by solving the Dirac-Weyl equation. We find that the peak value of TPA cross section is several orders of magnitude greater than that of conventional semiconductor QDs as a result of a large number of TPA resonance occurred easily due to the slight difference of energy level spaces in conduction and valence bands. Due to the quantum size effect, there is a red shift for the absorption peak and the magnitudes of the TPA coefficient increase with the increase of GQD’s radius. The QDs’ edge and electron relaxation rate can also tune the TPA. These theoretical analyses are of much importance to the applications based on two-photon fluorescence imaging as well as academic interest.

## Theory

**Figure 1:**
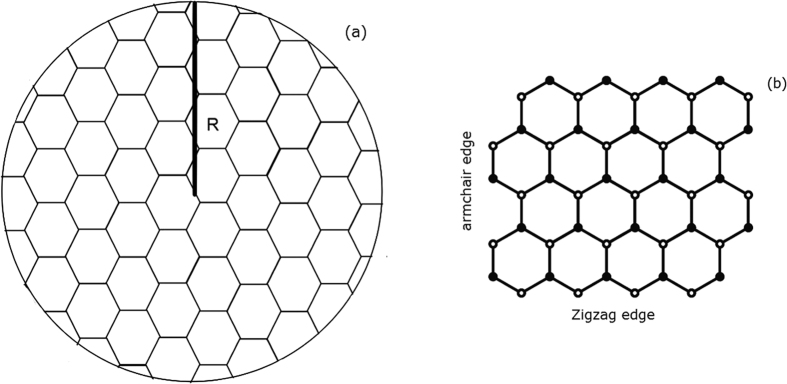
An isolated monolayer circular quantum dot (**a**) with armchair or zigzag dege (**b**).

We consider an isolated circular quantum dot with radius *R* made of monolayer graphene in Fig. 1. The GQD’s edge may be zigzag or armchair. The Dirac-Weyl Hamiltonian for low-energy electron states in GQD, in the absence of external field, reads


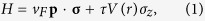


and the Dirac equation is *Hψ*(*r, θ*) = Εψ(*r, θ*) in cylindrical coordinates with the wave function being a two-component spinor, *ψ*(*r, θ*) = [*ψ*_1_(*r, θ*), *ψ*_2_(*r, θ*)]^T^. *v*_*F*_ is the Fermi velocity, and **σ** = (**σ**_*x*_, **σ**_*y*_) are Pauli matrices, which takes into account contributions of two different graphene sublattices. The parameter *τ* takes the two values ±1 distinguished the two valleys *K* and *K*′. We assume that a mass-related potential energy *V*(*r*) is coupled to the Hamiltonian^22^. The mass in the dot is zero, but trends to infinity at the edge of the dot, i.e. *V*(*r*) = 0 for *r* <* R*, and *V*(*r*) → ∞ for 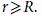. Therefore the Klein tunneling effect at the interface between the internal and external regions of the dot can be avoided and, carriers will be confined. Since the operator for the total angular momentum is conserved quantity, [*H, J*_*z*_] = 0, the two-component wave function has the form


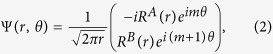


where *m* the angular momentum quantum number. Plugging this expression into the Dirac equation and decoupling the system of differential equations, we can obtain two differential equations in *K* valley,


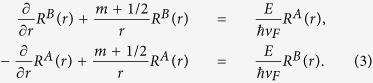


We employ the finite difference method to solve the above two differential equations. In consideration of the boundary conditions *R*^*A*^ (*r* = *R*) = *R*^*B*^ (*r* = 0) = 0 for zigzag edge and *R*^*A*^ (*r* = 0) = *R*^*B*^ (*r* = 0) = *R*^*A*^ (*r* = *R*) = *R*^*B*^ (*r* = *R*) = 0 for armchair edge^23^, the forward-difference to component *A* and backward-difference to component *B* are adopted in zigzag edge while central difference for armchair edge. Dispersing equation (3) and then diagonalizing the matrix, we can easily get the eigenvalues and eigenvectors, i. e. energy levels and wave functions of electron in GQDs.

Two-photon absorption is a process wherein two photons are absorbed simultaneously from the initial state through one intermediate state to the final state. The two-photon generation rate with incident light frequency *ω* can be represented in second-order perturbation theory with respect to the electron-photon interaction as^24^


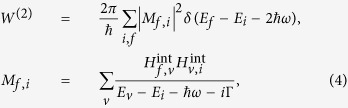


where *E*_*i*_, *E*_*f*_, *E*_*v*_ represent the energies of the initial, final and intermediate states of an electron, respectively. *H*^*int*^ = (*ev*_*F*_/*c*)**A·**σ describes the electron-photon interaction and **A** = *A***e** is the vector potential of the light wave with the amplitude *A* and the polarization vector **e**, and Γ is the relaxation energy in excited state. Based on the electron energy states derived above, the expression of matrix elements for one-photon transition from initial state |*i*〉 to intermediate state |*v*〉 in *K* valley can be written as


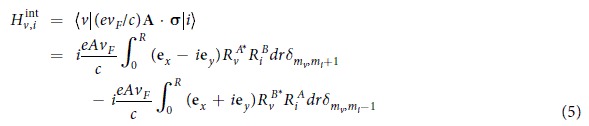


where **e**_*x*_, **e**_*y*_ are the Cartesian components of the polarization vector **e**, δ_*i,j*_ is the Kronecker delta function. Similar matrix elements for transition from intermediate state to final state can be obtained as well. It is easily found that a two-photon transition can only occur from initial state |*i*〉 via intermediate state |*v*〉 to final state | *f* 〉 for which the angular momentum quantum number of the electron satisfy the relations *m*_*v*_ − *m*_*i*_ = ±1 and *m*_*f*_ − *m*_*v*_ = ±1. These are the selection rules for a two-photon transition. The TPA coefficient β for an ensemble of QDs is related to the two-photon generation rate *W*^(2)^ by


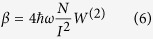


where *N* is the GQDs concentration and *I* is the incident radiation intensity 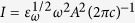, and ε_ω_ is the dielectric constant of the material at the light frequency. In most experiments, TPA is measured in terms of TPA cross section σ_2_. which is defined as


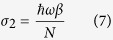


## Results and Discussion

Following the expressions derived above, we perform the calculations to predict the two-photon absorption spectra for monolayer GQDs with the shape of circular. Hereinafter calculations and discussions are carried out using the following parameters: *v*_*F*_ = 10^6^ m/s, *N* = 3 × 10^24^ m^−3^, Γ = 30 meV, *ε*_*ω*_ = 3.

**Figure 2:**
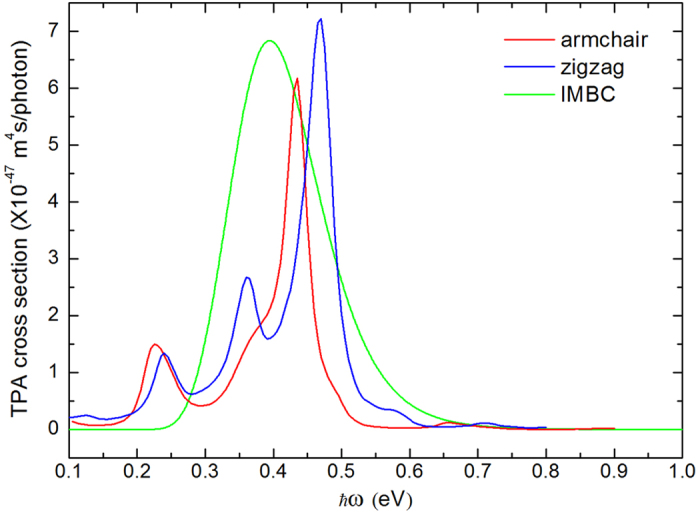
TPA spectra for circular GQDs (*R* = 2 nm) with three different boundary conditions: armchair, zigzag, and IMBC.

**Figure 3:**
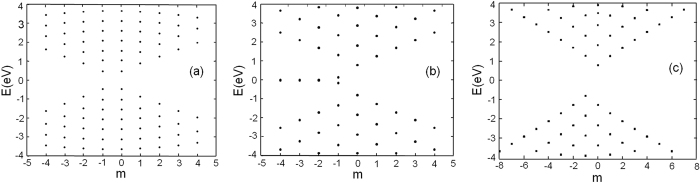
Energy levels of circular GQDs as a function of angular momentum label *m* for (**a**) armchair, (**b**) zigzag and (**c**) IMBC.

Here, by applying the TPA model presented in the previous section, we distinguish the different edges and simulate the TPA spectra in Fig. 2. The theoretical curve using IMBC is also inserted for comparison, in which the actual edge, zigzag or armchair, is ignored^21^. It is found that the TPA cross sections for all three different boundary conditions are in the order of 10^−47 ^m^4^s/photon at the absorption peaks. Compared the curve for armchair edge with that for zigzag, it is consistent that there are evident absorption peaks appeared when incident photon energy is about 0.2~0.6 eV (wavelength 2070~6212 nm), namely in infrared region. And the highest peak locates at 2856 nm and 2643 nm for GQDs with armchair and zigzag edge, respectively. The slight discrepancy results from the distinct electronic states, which are shown in Fig. 3(a–c). Different energy spectra of electron lead to different TPA spectra. We can see that the symmetry of energy spectra is different in some certain. For zigzag boundary condition, there are degenerate zero energy states appeared in band gap when angular momentum quantum number *m* is negative, which are the surface states. The energy spectrum for armchair is right-and-left symmetrical and *m* = 0, 1 are the symmetry axis. For IMBC, the energy levels in conduction band are of bilateral symmetry by 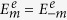, while in valence band the symmetry is 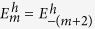. The TPA cross section values of both our theoretical calculations and experimental measurements in related materials are summarized in Table1 for comparison. The TPA cross section of GQDs is nearly 8 orders of magnitude greater than that of conventional semiconductor QDs in infrared region and 3 orders of magnitude greater in visible band^24^,^25^. The giant discrepancy is caused by the tiny changed energy level spaces in GQDs, which can be seen obviously in Fig. 3(a–c). It means that a large number of two photon transition resonance can occur easily in GQDs. While in conventional semiconductor QDs, energy levels spaces change a lot with the increase of quantum numbers^26^. So TPA resonances occur difficultly. Compared GQDs with other graphene-related materials, such as graphitic - C_3_N_4_ and Carbon - nanodot, the cross section in GQDs is still nearly 2–3 orders higher than that in graphitic - C_3_N_4_ and Carbon - nanodot in 750 and 720 nm^19^,^20^. It is plausible that the discrepancy may result from the non-uniform size of experiment samples. The size-dependence of GQDs will be discussed below. It is also possible that scattering effect takes effect in TPA measurement and weakens the two-photon absorption. Also, the lower electron relaxation energy 30 meV (~20 fs) we chose in excited state will also raise the TPA value.

**Figure 4:**
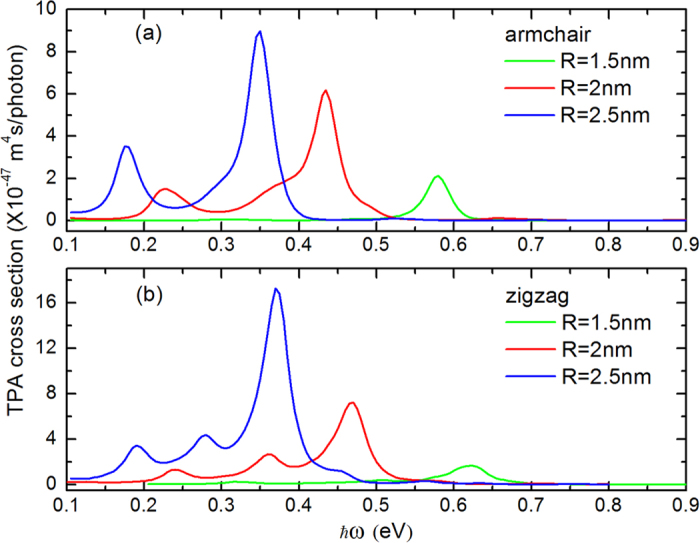
TPA cross section of three different sized QGDs with edge of (**a**) armchair and (**b**) zigzag plotted as a function of incident photon energy.

By applying the TPA model presented in the previous section, the TPA spectra for three different size GQDs with armchair and zigzag edges are shown in Fig. 4. All the transitions satisfied selection rules for 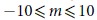 are included in our calculation. We can find that with the increasing of the GQD’s radius, there is a red shift for the absorption peak. Also, with the increase of *R*, the magnitude of TPA cross section increases too. This is owing to the fact that as the consequence of quantum size effect, energy differences become smaller and density of states increase when *R* increases^21^. The bigger the GQD is, the lager the density of state is. Therefore, more transitions can occur.

**Figure 5:**
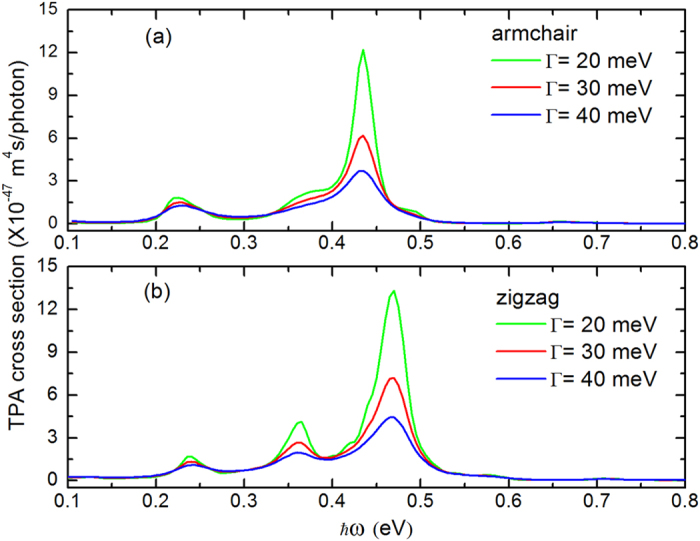
TPA spectra for different electron relaxation energy for 2 nm sized GQDs with the edge of (**a**) armchair and (**b**) zigzag.

An other parameter in the expression of TPA coefficient for circular GQDs is the relaxation energy Γ in excited state. The TPA spectra for different relaxation energies 20, 30, 40 meV, which correspond to dephasing times 30, 20, 15 fs, are shown in Fig. 5. We can find that the lower the electron relaxation energy is, the higher the absorption peak is. Wheras there is no shift accompany happened to the absorption peak. These results can be explained as follows: for GQDs with a fixed size, the magnitude of TPA coefficient is determined by the factor |*M*_*f*,*i*_|^2^ while the position of absorption is determined by Dirac delta function in equation (4). When TPA resonance occurs, 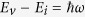 and 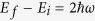, Dirac delta function, i. e. the position of peak is independent on relaxation energy. At this moment, we can deduce 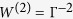, that is to say, the peak value of TPA cross section is inversely proportional to the square of relaxation energy.

## Conclusion

In conclusion, by solving the Dirac-Weyl equation under finite difference method, we have obtained the energy spectrum of a circular GQD with both armchair edge and zigzag edge. There appears the surface states in zigzag edged GQDs and the energy spectra are of symmetry in some extent. On the basis of energy levels and electronic states, the expressions for the size-dependent two-photon absorption cross section have been deduced. The TPA spectra reveal that the peak value of TPA cross section is several orders of magnitude greater than that of conventional semiconductor QDs. With the increase of GQD’s radius, there is a red shift for the absorption peak and the magnitudes of the TPA cross section increase too. And the TPA peak value and position can be tuned by the size and edge of the GQDs. The electron relaxation energy in excited state will not change the position of TPA resonance but the peak value. These theoretical analyses are of great importance to applications based on two-photon fluorescence imaging as well as academic interest.

## Additional Information

**How to cite this article**: Feng, X. *et al*. Giant Two-photon Absorption in Circular Graphene Quantum Dots in Infrared Region. *Sci. Rep.* **6**, 33260; doi: 10.1038/srep33260 (2016).

## Figures and Tables

**Table 1:** Calculated values of TPA cross section in circular GQDs and experimental data in other related materials for comparison.

Materials	Size (nm)	wavelength (nm)	σ_2_ (GM)	
			Experiment	Theory
Circular GQDs(armchair)	4	2856		6.2 × 10^11^
	4	800		2.7 × 10^7^
	4	780		3.2 × 10^7^
	4	750		8.5 × 10^6^
	2	720		4.3 × 10^5^
	4	532		5.0 × 10^5^
Circular GQDs(zigzag)	4	2643		7.2 × 10^11^
	4	800		2.3 × 10^7^
	4	780		8.7 × 10^6^
	4	750		1.3 × 10^7^
	2	720		6.5 × 10^5^
	4	532		5.1 × 10^5^
Graphitic–C_3_N_4_^19^	2~6	750	2.8 × 10^4^	
Carbon–nanodot^20^	1~3	720	1.32 × 10^3^	
CdS nanosphere^24^	4.4	800	4.5 × 10^3^	
CdS nanorod^25^	*d* = 4.4, *L* = 43	800	2.1 × 10^5^	
